# A 10-year clinicopathological analysis of ovarian lesions in a tertiary hospital in Southern Nigeria

**DOI:** 10.4314/ahs.v23i1.48

**Published:** 2023-03

**Authors:** Aniekan M Abasiattai, Chukwuemeka C Nwafor, Ntiense M Utuk

**Affiliations:** 1 Department of Obstetrics/Gynaecology, College of Health Sciences, University of Uyo; 2 Department of Pathology, College of Health Sciences, University of Uyo

**Keywords:** Ovary tumours, ovarian malignancy, malignant tumours, benign tumours, histopathology Uyo

## Abstract

**Background:**

Ovarian tumours are the most lethal of all gynaecological cancers and they are usually diagnosed in advanced stages when the prognosis is very poor.

**Objective:**

To determine the pattern of ovarian lesions, their frequency, presentation, and associated clinical symptoms in Uyo, Nigeria.

**Methods:**

A 10-year retrospective study of all ovarian specimens that were surgically removed and histologically diagnosed.

**Results:**

The patients were between the ages of 5 and 73 years with median age of 34.1 years. Benign tumours occurred most commonly among the 20–39-year age group (31.3%) while malignant tumours were predominant among those aged 50-69 years (10.0%). Surface epithelial tumours (45.4%) were the most common neoplastic tumours while the mature cystic teratoma (33.2%) was the most common tumour overall. Surface epithelial malignancies accounted for 70.6% of all ovarian malignancies and the serous cyst adenocarcinoma (10.2%) was the most common surface epithelial tumour as well as the most common malignant tumour.

**Conclusion:**

There has been an increase in the number of malignant ovarian specimens in our centre. Though surface epithelial tumours were the most common category of ovarian tumours, overall, the mature cystic teratoma was the most common tumour. Serous cyst adenocarcinoma was the most common surface epithelial tumour and the most common malignant tumour.

## Introduction

Lesions of the ovary constitute a significant source of morbidity and mortality particularly in the developing world. They are classified into non neoplastic lesions (inflammatory and functional cysts), and neoplastic lesions which include benign, borderline and malignant ovarian tumours 1.

The frequency of benign ovarian tumours varies with age and the most common benign ovarian tumours in young and elderly women are the germ cell and surface epithelial tumours 2. Though the majority of benign ovarian tumours are asymptomatic, they can cause menstrual abnormalities, pressure symptoms like frequency of micturition, dyspareunia, abdominal pain which may be severe when torsion occurs and signs of peritonitis if they rupture 3.

Ovarian tumours represent about 30% of all cancers of the female genital tract and 4% of cancers in women 4. They are the most lethal of all gynaecological cancers partly due to their insidious presentation and anatomic location as well as their intrinsic histological and molecular heterogenicity 5. In addition, they are the cause of more deaths than every other malignancy affecting females 6. Ovarian cancers have been described as silent killers as they are often difficult to detect until they are advanced in stage or size as their symptoms are non-specific, vague and insidious 7.

There are an estimated 192,000 cases of ovarian cancer per year worldwide and unfortunately, the majority (75%) are diagnosed with advanced disease (stages III and IV) when the 5-year survival rate is less than 20% 5. There is great variation in the geographic distribution of ovarian cancer with the highest incidence reported to be in North America and Europe especially the Nordic countries and the United Kingdom 5. Interestingly, a previous report from this centre 8 and indeed other centres in Nigeria 9–11 reveal that ovarian cancer is the second most common gynaecological cancer in these environments after cancer of the cervix.

Since the establishment of our centre, there has been no known study on ovarian lesions. Hence, this study aims to determine the pattern of ovarian lesions, their frequency, presentation, and associated clinical symptoms in the University of Uyo Teaching Hospital.

## Material and Methods

This is a retrospective study of all ovarian specimens (OS) that were surgically removed and histologically diagnosed in the Obstetrics/Gynaecology and Histopathology Departments of University of Uyo Teaching Hospital (UUTH) respectively over a 10 year period (from January 1, 2010 to December 31, 2019). UUTH, a 500-bed hospital is the only tertiary hospital in Akwa Ibom State and also serves as a referral centre for the neighbouring states. The histopathology laboratory in UUTH is the main facility where histopathology services are rendered in Akwa Ibom State and as such render's services to the host hospital and many privately owned and public hospitals. These OS included cystectomies, oophorectomies, incisional biopsies and hysterectomies. Following laparotomies, these OS were immediately fixed in 10% buffered formalin, auto-processed, and paraffin embedded sections were taken and stained with hematoxylin and eosin. Special stains were used when necessary.

Data were extracted from the patients' case files and theatre registers. Other major sources of information were histopathological departmental registers, patient request forms, and duplicate copies of histology reports of all cases. Information extracted included the age of patients, major symptom/s prior to presentation, duration of symptom/s before presentation, laterality of the tumour, clinical diagnosis, maximum diameter and weight of the specimen, type of specimen received and histologic diagnosis. The OS were classified histologically into cystic lesions, inflammatory lesions and neoplastic lesions. The neoplastic lesions were further sub-classified using the World Health Organisation's (WHO) international classification of ovarian tumours (into surface epithelial, germ cells and sex cord stromal tumours) 12. Data were analysed using predictive analytical software, version 17 (IBM, SPSS Inc., Chicago, IL, USA).

Simple frequencies were determined for categorical variables and the mean was evaluated for continuous data. Ovaries that were part of hysterectomy specimens performed due to non-ovarian lesion indications, like those due to endometrial lesions, myometrial lesions and cervical lesions were excluded. OS from incidental ovarian cystectomies performed during elective caesarean sections (CS) were included. Few reports with ambiguous conclusions were excluded. Also excluded were OS reports with any of the major identification parameters (such as age and histologic diagnosis) missing. Six reports in all were excluded.

Since the aim of our study was to document pathological ovaries seen, those from hysterectomies of non-ovarian indications were excluded because they are almost always normal. In addition, most hysterectomies in our centre are performed for myometrial and endometrial lesions.

## Results

A total of 7,028 histologic specimens were received in the department of Histopathology during the 10 years under review, while ovarian tissues (excluding ovaries that were part of hysterectomies performed for other reasons like, cervical, endometrial and myometrial lesions) were 160 accounting for 2.3% of all histopathologic specimens.

Over the years there had been a mild increase in the number of specimens sent for histologic examination. Two peaks (year 2012 and 2019) were observed to have the highest number of ovarian specimens. There had been a marginal increase in the number of malignant ovarian specimens since the year 2014, as shown in table 1. Year 2019 recorded the highest number of malignant ovarian lesions though, that was the year also with the highest number of specimens sent in for examination.

**Table 1:** Yearly pattern of ovarian specimens in relation to ovarian malignant lesions

Year	Total specimens	All ovarian specimens	Malignant ovarian lesion
2010	530	7	0
2011	638	9	4
2012	650	23	2
2013	782	19	2
2014	595	11	2
2015	722	13	5
2016	685	14	4
2017	762	17	5
2018	753	18	4
2019	911	29	6
Total	7028	160	34

Table 2 shows t he presenting complaints, duration of symptoms prior to presentation, clinical diagnoses, laterality of the ovarian lesions/mass and procedure performed by the gynaecologist. The earliest a patient presented was within two days of onset of symptoms, while the longest interval before presentation was 15 years (180 months), with a mean duration of 24.2 ±35.15 months. A significant proportion of the patients (22.5%) presented at least more than one year after onset of symptoms. The minimum diameter of ovarian specimens seen was 2cm, while the widest diameter seen was 47cm. The weight of ovarian specimens ranged from 100g to 40000g with a mean weight of 2361g. Right ovary specimens accounted for most (30.6%), while cystectomy (58.8%) was the commonest surgical procedure performed by the gynaecologist. Ovarian tumour or ovarian cyst (47.5%) was the most commonly made provisional diagnosis.

**Table 2:** Pre histologic characters of the ovarian specimens

Various Characteristics	Frequency	Percentage (%)
Clinical Diagnoses Ovarian tumor/cyst Ovarian torsion Ovarian malignancy Meigs syndrome Others Not stated	76 14 14 6 8 42	47.5 8.8 8.8 3.8 4.8 26.3
Laterality Left Right Bilateral Not stated	34 49 22 55	21.3 30.6 13.8 34.3
Surgical procedure Cystectomy Oophorectomy Hysterectomy Incisional biopsy Not stated	94 30 26 3 7	58.8 18.8 16.3 1.7 4.4
Duration of symptoms before presentation < 3 months 4–6 months 7–11 months 1 year and above Not stated	30 13 7 36 74	18.8 8.1 4.4 22.5 46.2
Presenting complaints Lower abdominal pain Abdominal swelling Abdominal mass Weight loss Removed during elective CS Per vagina bleeding Amenorrhea Vomiting Others	75 57 23 10 7 6 6 6 46	46.9 35.6 14.4 6.3 4.4 3.8 3.8 3.8 27.6

Others in clinical diagnoses include each of the following: ectopic pregnancy, intra-abdominal malignancy, Poly cystic ovarian syndrome, ovarian abscess, endometriosis, and uterine sarcoma. Other presenting complaints included: Primary infertility, dysmenorrhea, breathlessness, Irregular menses, bleeding per rectum, leg swelling, constipation, lower urinary tract obstruction, vaginal discharge and each of the following: post-menopausal bleeding, sprouting of pubic hair in a school age child, breast enlargement, easy satiety, acute abdomen, melena stool and found incidentally during a myomectomy.

All the specimens were from phenotypically and genetically females. The youngest patient was 5 years old and the oldest 73 years old with a mean age of 34.13±13.78. Generally, age groups 20-29 years, closely followed by 30-39 years accounted for more than 66.0% of cases, while benign neoplastic lesions were the commonest histologic category of specimens seen. For the malignant lesions, age group 50-59 years alone accounted for 32.4% of cases as shown in table 3. Neoplastic ovarian lesions (benign, borderline and malignant lesions) accounted for 67.5% of all diagnoses made histologically with malignant ovarian tumours consisting of 21.2%.

**Table 3:** Age group distribution of ovarian specimens in relation to major histologic category of lesion

Histologic category	< 19 years	20–29 years	30–39 years	40–49 years	50–59 years	60–69 years	70–79 years	Total	Percentage (%)
Benign neoplastic lesions	5	28	22	7	3	5	1	71	44.4
Cystic non neoplastic lesions	1	19	18	1	-	-	-	39	24.4
Malignant neoplastic lesions	4	5	4	4	11	5	1	34	21.2
Inflammatory lesions	3	2	6	-	-	1	-	12	7.5
Borderline malignant lesions	-	-	1	1	1	-	-	3	1.9
Ectopic normal tissue	-	-	1	-	-	-	-	1	0.6
Total	13 (8.1%)	54 (33.8%)	52 (32.5%)	13 (8.1%)	15 (9.4%)	11 (6.9%)	2 (1.2%)	160 (100%)	100%

P = 0.000

Neoplastic ovarian tumours made up 108 (67.5%) of the specimens. Tumours of surface epithelial origin accounted for 45.4%, closely followed by tumours of germ cell origin (40.7%). The most common benign tumours of epithelial origin were the serous cyst adenomas (8.3%), while serous cyst adenocarcinomas were the most common malignant tumour of epithelial origin (10.2%) and also the most common malignant tumour overall. Mature teratomas (MT) were not only the most predominant germ cell tumour, but were also the most common tumour seen overall accounting for 33.2% of all ovarian tumours. Involvement of both ovaries by the same pathology was observed in a case of serous cyst adenocarcinoma, endometrioid carcinoma and serous cyst adenoma. In 2 situations, bilateral MT was observed (table 4).

**Table 4:** Histopathologic diagnoses of neoplastic ovarian specimens

Histopathologic diagnoses	Frequency	Percentage
**Benign surface epithelial lesions** **(n=22)** Serous cyst adenoma Mucinous cyst adenoma Mucinous cyst adenofibroma Serous cyst adenofibroma Left M C A and right Brenner's tumor	9 8 1 3 1	8.3 7.4 0.9 2.8 0.9
**Borderline surface epithelial (n=3)** Borderline mucinous cyst adenoma	3	2.8
**Malignant surface epithelial lesions** **(n=24)** Serous cyst adenocarcinoma Mucinous cyst adenocarcinoma Endometrioid carcinoma Transitional cell carcinoma Malignant mixed mullerian tumor	11 9 2 1 1	10.2 8.3 1.9 0.9 0.9
**Benign sex cord stromal lesions (n =** **7)** Fibrothecoma Adult granulosa cell tumor Fibroma Thecoma	3 2 1 1	2.8 1.9 0.9 0.9
**Malignant sex cord stromal tumors** **(n = 3)** Adult type granulosa cell tumor Fibrosarcoma	2 1	1.9 0.9
**Benign germ cell tumors (n = 41)** Mature teratoma Struma ovarii Left corpus luteum cyst and right M T	36 2 3	33.2 1.9 2.8
**Malignant germ cell tumors (n =3)** Immature teratoma Malignant transformation of a M T	2 1	1.9 0.9
**Other benign tumors (n = 1)** Leiomyoma	1	0.9
Metastatic tumors (n = 4) Non-Hodgkin's lymphoma Krukenberg tumor	2 2	1.9 1.9
**Total**	**108**	**100**

M T = Mature teratoma

The age range of patients with malignant ovarian tumours was between 5 years and 71 years with a mean age of 43.59 ±17.72. The sizes of malignant ovarian specimens ranged from 2cm to 47cm with a mean diameter of 16.48cm ±8.27. The mean weight of malignant ovarian specimens was 4402grams ±9009.27. The least number of malignant lesions was seen in age group 70-79 years (2.8%), while the highest was seen in age group 50-59 years (32.4%). The remaining cases were roughly equally distributed across the other age groups as shown in table 5. Surface epithelial origin malignancies accounted for 70.6% of all ovarian malignancies seen. Germ cell and sex cord stromal malignancies each accounted for 8.8% of malignant ovarian lesions respectively. Involvement of both ovaries by the same malignant pathology was observed in a case of serous cyst adenocarcinoma (in a patient aged 57 years) and endometrioid carcinoma (in a patient aged 15 years).

**Table 5:** Age group distribution of malignant ovarian tumours

Malignant diagnosis	Age group							
	<19	20–29	30–39	40–49	50–59	60–69	70–79	Total (%)
Serous cyst adenocarcinoma	-	1	-	1	6	2	1	11(32.4)
Mucinous cyst adenocarcinoma	1	1	1	2	2	2	-	9 (26.5)
Endometrioid carcinoma	1	1	-	-	-	-	-	2 (5.9)
Krukenberg tumor	-	-	-	-	2	-	-	2 (5.9)
Immature teratoma	2	-	-	-	-	-	-	2 (5.9)
Malignant adult type granulosa tumor	-	-	1	-	1	-	-	2 (5.9)
Non-Hodgkins lymphoma	-	2	-	-	-	-	-	2 (5.9)
Transitional cell carcinoma	-	-	1	-	-	-	-	1 (2.9)
Malignant mixed mullerian tumor	-	-	1	-	-	-	-	1 (2.9)
Malignant transformation of MT	-	-	-	-	-	1	-	1 (2.9)
Fibrosarcoma	-	-	-	1	-	-	-	1 (2.9)
Total	4 (11.8)	5 (14.7)	4 (11.8)	4 (11.8)	11 (32.4)	5 (14.7)	1 (2.8)	34 (100)

P = 0.133

Cystic lesions of the ovary were the most common non-neoplastic lesions seen in this study (75%). Corpus luteum cyst, closely followed by follicular cyst and simple cyst each accounted for 23.1%, 21.2% and 21.2% respectively as shown in table 6.

**Table 6:** Non neoplastic histologic diagnoses

Non neoplastic lesions	Frequency	Percentage
**Cystic ovarian lesions (n = 39)** Corpus luteum cyst Follicular cyst Simple cyst Corpus haemorrhagicum Luteoma of pregnancy Polycystic ovarian syndrome	12 11 11 3 1 1	23.1 21.2 21.2 5.8 1.9 1.9
**Inflammatory lesions (n = 12)** Abscess / oophoritis Ovarian torsion	7 5	13.5 9.5
**Ectopic normal tissue (n = 1)** Endometriosis	1	1.9
**Total**	**52**	**100**

## Discussion

This study reveals the pattern of ovarian lesions in our centre. The mean age of our patients was 34.1 years with benign tumours occurring most commonly among the 20-39 years age group, while malignant tumours were found predominantly among those aged between 50-69 years. These findings are consistent with those of other researchers 13,14, who have also documented these age groups to be the most commonly affected by benign and malignant neoplastic tumours in their series. Benign neoplastic lesions were in the majority as they accounted for 65.7% of the specimens. However, the proportions of benign and malignant tumours in our study (65.7% and 34.3%) were at variance with those from other centres where the percentages of benign and malignant tumours were 84.9% and 13.4% 1, 84.7% and 15.3% 2, 78.3% and 18.4% 4, and 83.8% and 14.4% 14 relatively. Available reports from Caucasian and Western countries also indicate that 75.0%-80.0% of ovarian tumours are benign, while malignant tumours account for the remaining 20.0%-25.0% 4. Hence, when compared with what obtains in the Western world and several centres in Asia, the proportion of ovarian tumours that are malignant in our environment is much higher.

Overall surface epithelial tumours were the most common ovarian tumours and were very closely followed by tumours of germ cell origin. This is in accordance with reports from several other researchers 1,13–16. However, not only were the mature teratomas which are benign germ cell tumours the most common ovarian lesions in our study, they were also more common than the total sum of all other benign tumours. Studies conducted in Nigeria and indeed other parts of Sub-Saharan Africa have also revealed the mature cystic teratomas to be the most common ovarian tumours in their various populations 2,17–20. This finding is completely at variance with what obtains in the North America, Europe and Asia where mostly serous cystadenomas or mucinous cyst adenomas, which are both benign surface epithelial tumours, are much more common 1,4,13–16.

Due to the retrospective nature of our study, the reason for the high preponderance of the dermoid cysts in African populations could not be discerned. However, this predisposition is likely to be due to racial factors as shown in a South African study19 where there was a predominance of germ cell tumours in blacks (52.0%) as opposed to Indians (19.0%), with benign cystic teratoma accounting for most of the cases.

Malignant tumours made up a significant proportion of all the specimens as they constituted a third of all lesions. Malignant surface epithelial tumours accounted for about 70% of all ovarian malignancies. The proportion of malignant surface epithelial tumours in our study is much higher than those recorded by several other authors 1,2,13,14. Furthermore, serous cystadenocarcinoma which was the most common malignant tumour in our study was also the most common surface epithelial lesion. Though, the benign serous cystadenoma is documented to be the predominant surface epithelial tumour and occurs much more commonly than its malignant form in most other series 1,2,13,14,18,21, the converse was the case in ours as its malignant form was much more common.

While literature shows that the majority of mucinous tumours are benign 13,16, with 20.0% being borderline and 5.0% invasive 13, in our series, the mucinous cysadenocarcinoma constituted 26.5% of all malignant ovarian tumours, was the second most common malignant ovarian tumour, occurred more commonly than its benign form, and occurred at the same frequency with the benign serous cystadenoma. This seems to suggest that in contrast to what is in available literature, the malignant mucinous cyst adenocarcinoma is not uncommon in our environment.

It is also worrisome that in our study, not only have ovarian malignancies increased in number over the last year, but they were also found in adolescents, half of whom had the very virulent malignant surface epithelial tumours. In a study conducted by Ahmed et al in Dhaka, Pakistan 1, all cases of ovarian malignancy that occurred in patients under 20 years of age were germ cell in origin. Malignant germ cell tumours have a better prognosis when compared to malignant surface epithelial tumours and they are often cured with conservative surgery and chemotherapy with preservation of fertility 22.

Functional cysts (corpus luteum, follicular and simple cysts) were the most common non-neoplastic cysts in our series. This is similar to the findings of Amin et al in Lagos, Nigeria 17 and Neelgund et al in Pondicicherry, India^21^ but different from those of Naik et al in Sattur, India 14 were chocolate cysts where much more common. Functional or physiological cysts are large versions of cysts that form in the ovary during the normal menstrual cycle. They are asymptomatic, resolve spontaneously and often do not require any treatment 21.

The common symptoms associated with ovarian lesions were lower abdominal pain, abdominal swelling and an abdominal mass. This is similar to what obtains in other reports 14. The symptoms of ovarian tumours are non-specific and vague and there are no distinguishing symptoms that differentiate benign from malignant ovarian tumours 23. Indeed, malignant ovarian tumours are frequently asymptomatic until they are advanced in stage^14^. Hence, it is imperative clinicians maintain a high index of suspicion in-order to detect ovarian malignancy early; ensure prompt treatment with an ultimate increase in survival from the disease and a decrease in treatment related morbidity.

This study was retrospective in nature and was conducted in a tertiary health facility, thus it may not be representative of what obtains in the community.

In conclusion, there has been an increase in the number of malignant ovarian specimens in our centre. Neoplastic ovarian tumours were the commonest lesions; however, the percentage of malignant tumours was high which is at variance with findings from studies in Asia and the Western world. Though surface epithelial tumours were the most common category of ovarian tumours, overall, the mature cystic teratoma was the most common tumour. Not only was the serous cyst adenocarcinoma the most common surface epithelial tumour, it was also the most common malignant tumour in the study. Thus, practicing clinicians must maintain a high index of suspicion in order to detect ovarian malignancy early and offer prompt treatment.
